# Combined and Modified Gibson and Ilioinguinal Approaches in Type II + III Internal Hemipelvectomy for Periacetabular Tumors

**DOI:** 10.3389/fonc.2022.934812

**Published:** 2022-07-13

**Authors:** Xin Hu, Minxun Lu, Jie Wang, Longqing Li, Li Min, Chongqi Tu

**Affiliations:** ^1^ Department of Orthopedics, Orthopedic Research Institute, West China Hospital, Sichuan University, Chengdu, China; ^2^ Department of Model Worker and Innovative Craftsman, West China Hospital, Sichuan University, Chengdu, China

**Keywords:** hemipelvectomy, 3D-printed, Gibson approach, iliofemoral approach, ERAS

## Abstract

**Background:**

The routine iliofemoral approach and its modifications in type II+III resection require extensive skin incision and massive periacetabular muscle detachment, leading to prolonged hospital stay, increased complication incidence, and impaired lower limb function. Under the management of an enhanced recovery after surgery (ERAS) protocol, a combined and modified Gibson and ilioinguinal (MGMII) approach was used to avoid unnecessary soft tissue trauma during tumor resection and therefore advantageous to patients’ return to normal life.

**Methods:**

Twenty-five patients with type II + III (including type II) periacetabular tumors who underwent reconstruction with 3D printed customized endoprostheses at our center between January 2017 and March 2019 were included in this study. There were 13 cases using MGMII approach and 12 cases using iliofemoral approach. The operation duration and blood loss were assessed by chart review. The surgical margin was evaluated by the histopathological studies. The reconstruction accuracy, the abductor muscle strength, the 1993 version of the Musculoskeletal Tumor Society (MSTS-93), the Harris Hip scores (HHS), and the limp score were evaluated. Complications were recorded after reviewing the patients’ records.

**Results:**

The operative duration and blood loss in MGMII group were shorter than those in the iliofemoral group, but the postoperative hemoglobin was slightly higher than that in the iliofemoral group. The MGMII group had stronger postoperative hip abductors, better functional restoration, and relatively fewer patients with higher limp scores. No complication was observed in the MGMII group. In the iliofemoral group, three patients encountered wound healing delay, and one patient suffered deep infection.

**Conclusions:**

The MGMII approach can better expose the posterior column of the acetabulum, especially the ischial tuberosity, which is beneficial for avoiding tumor rupture during resection. The MGMII approach also helps to preserve residual muscle function, such as the origin of the gluteus medius, while ensuring the extent of resection.

## Introduction

Since Enneking and Dunham described hemipelvic limb salvage surgery and classified the resections into four basic types in 1970s, numerous internal hemipelvectomies have been reported. Type II+III (including type II) resection, with an incidence ranging 43%–90%, composed the majority of this group (1–4). Reconstruction after these internal hemipelvectomies is of great necessity, and endoprosthesis reconstruction can rebuild functionalized hip after periacetabular tumor removal, which has been accepted as the mainstream approach (5). To accomplish this highly technical procedure, various approaches have been introduced and modified such as the iliofemoral approach, the ilioinguinal approach, and the Gibson approach (3, 6–9).

The iliofemoral approach and its modifications are the most common surgical approaches, which can access the sciatic notch both internally and externally by separating the abdominal muscles, iliopsoas, and gluteus from the ilium (10–12). However, there are also several limitations while performing type II+III resection *via* these approaches. First of all, when the tumor invades a deeper area such as ischial tuberosity and its adjacent structures, the view of the operation field may be limited due to unfavorable tumor location or tumor size (3). In such a scenario, performing en bloc resection can be difficult and technically demanding, and the tumor can easily rupture with disastrous consequences. Additionally, limb functional impairment might be associated with the influence of the gluteus, iliacus, and femoral nerve (10, 13, 14). Moreover, using these approaches is prone to wound complications (15–19). The ilioinguinal approach was introduced by Letournel in 1961. As a standard approach to access anterior column, it is difficult to expose the surgical field of posterior column (20). Moreover, the routine ilioinguinal approach requires extensive blunt dissection of the iliacus to expose iliac fossa, which might be excessive for a type II+III resection. In contrast, the Gibson approach, described by Alexander Gibson in 1950, provides direct access to the outer surface of the posterior column and posterior wall and indirect access to the superior wall and quadrilateral surface, without impairing the muscles that are not detached from an extensive iliac origin such as the iliacus (21). Currently, the Gibson approach has been widely accepted in total hip arthroplasty to offer similar or superior functional restoration and a lower complication rate to other approaches (22). It is also generally considered as an additional approach in orthopedic oncology field (23).

With the popularization of the concept of enhanced recovery after surgery (ERAS) program (24), much attention has recently been given to modified surgical techniques that result in effective hemorrhage control, reduced postoperative pain, lower bedrest requirements, and shorter hospital stays (25–29). The modification of surgical approach is considered to be of major importance in the ERAS strategies. However, since each of the surgical approaches have their own pitfalls, oncologists may struggle to meet the ERAS needs of cancer patients with the sole-incision approach. In order to benefit patient’s enhanced recovery, we modified the Gibson approach and ilioinguinal approach and combined them to access the tumor lesion from both sides for adequate exposure and little disruption to major periacetabular muscles. Herein, in this study, two surgical approaches used in hemipelvic replacement with 3D-printed custom-made endoprosthesis, namely, MGMII approach and iliofemoral approach, are compared in terms of surgical convenience and accuracy, functional recovery status, and short-term complications aimed at the identification of better surgical approach.

## Patients and Methods

### Patient Selection and Demographics

From January 2017 to March 2019, our center admitted 31 patients with II+III (including type II) benign aggressive or malignant tumors. Hemipelvic replacement surgery is recommended if the assessment suggests that limb salvage surgery can achieve adequate surgical margins and that satisfactory function can be preserved by reconstruction after resection.

Patients who met the following criteria were included: (1) according to the Enneking and Dunham classification of pelvic tumors, tumors located in zones II + III (patients with types I + II + III were excluded); (2) reconstruction with 3D-printed custom-made integrated endoprostheses; (3) had a definite pathological diagnosis; (4) had complete data, including clinical records, imaging, and pathological reports; and (5) had a minimum follow-up of 24 months after surgery. Patients with incomplete follow-up data, patients with serious osteoporosis, patients with deformities of the lower limbs, patients with metal implant allergy, patients with gluteus medius invaded by tumors were excluded.

From a total of 31 patients with II+III tumors, 6 patients were excluded for the following reasons: 3 patients were lost to follow-up, and 3 patients opted for reconstruction with a modular hemipelvic prosthesis due to the time taken to accept a customized hemipelvic prosthesis or financial constraints. The remaining 25 patients were included in the study. Specific information about sex, age, the tumor type, grade (30), and chemotherapy is shown in **Tables 1**, **2**. This study was approved by the ethical committee of our institution. Written informed consent to participate in this study was obtained from all patients.

**Table 1:** Demographics of the 25 patients treated with 3D-printed custom-made integrative hemipelvic endoprostheses *via* two approaches.

Patient	Age (years)	Gender	BMI	Follow-up (months)	Operative duration (minutes)	Blood loss (ml)	PRBC transfusion (units)	Preoperative hemoglobin (g/L)	Postoperative hemoglobin (g/L)	Complications
1	53	F	19	51	210	1,900	8.0	114	102	
2	40	M	24	50	180	2,300	/	142	93	
3	43	F	20	47	240	2,400	10.0	129	99	DWH
4	16	M	27	33	560	2,000	6.0	125	101	
5	67	M	29	38	420	2,500	8.5	137	105	DWH
6	38	F	20	31	360	1,000	5.5	122	114	Infection
7	44	M	24	45	270	3,100	7.5	143	98	
8	38	M	23	28	360	1,000	/	136	116	DWH
9	46	M	25	44	300	3,500	11.0	121	89	
10	35	F	20	41	330	3,200	7.0	146	100	
11	25	F	23	36	270	2,400	7.0	127	102	
12	57	M	22	42	390	2,900	10.0	119	95	
13	40	F	27	49	170	1,700	/	156	115	
14	68	M	27	47	360	2,000	3.0	135	101	
15	57	M	21	46	240	1,500	7.5	114	117	
16	20	M	26	43	250	1,500	5.0	118	108	
17	48	M	27	32	300	2,000	11.0	98	104	
18	25	M	23	43	270	1,800	8.0	118	112	
19	34	F	23	35	240	1,000	/	120	93	
20	26	M	22	29	180	800	/	133	113	
21	50	F	20	46	260	1,300	/	139	120	
22	35	M	21	40	320	2,500	8.0	135	110	
23	23	M	26	34	210	1,400	5.0	112	102	
24	42	M	22	24	230	1,700	/	144	99	
25	23	F	20	39	280	2,200	9.0	116	98	

BMI, body mass index; MGMII, modified Gibson and modified ilioinguinal; DWH, delayed wound healing; PRBC, packed red blood cells.

**Table 2:** Preoperative oncologic characteristics of 25 patients treated with 3D-printed custom-made integrative hemipelvic endoprostheses *via* two approaches.

Patient	Approach	Diagnosis	Enneking staging (16)	Tumor location^a^	Posterior acetabular column involvement	Ischial tuberosity bone destruction	Tumor length(cm)	Tumor width(cm)	Tumor height(cm)	Neoadjuvant chemotherapy	Postoperative chemotherapy recovery time(days)
1	Iliofemoral	Osteosarcoma	III	PII+III	No	No	6.7	3.4	9.1	Two cycles	27
2	Iliofemoral	Chondrosarcoma	IIB	PII+III	Yes	No	6.1	2.4	5.5	No	Not applicable
3	Iliofemoral	Chondrosarcoma	IIB	PII+III	Yes	No	5.1	2.5	5.8	No	Not applicable
4	Iliofemoral	Ewing sarcoma	IIB	PII+III	No	No	9.6	5.8	7.6	Two cycles	26
5	Iliofemoral	Chondrosarcoma	IIB	PII+III	Yes	No	14.1	8.3	10.5	No	Not applicable
6	Iliofemoral	Malignant peripheral nerve sheath tumor	IIB	PII+III	Yes	No	10.1	6.9	9.2	No	Not applicable
7	Iliofemoral	Ewing sarcoma	IIB	PII+III	No	No	8.3	2.9	3.3	Two cycles	21
8	Iliofemoral	Chondrosarcoma	IIB	PII+III	No	No	7.4	5.2	6.4	No	Not applicable
9	Iliofemoral	Renal clear cell carcinoma	/	PII+III	No	No	9.9	7.6	7.6	No	Not applicable
10	Iliofemoral	Chondrosarcoma	IIB	PII+III	No	No	10.0	4.9	7.2	No	Not applicable
11	Iliofemoral	Osteosarcoma	IIB	PII+III	No	No	8.5	6.1	5.4	Two cycles	23
12	Iliofemoral	Solitary plasmacytoma	IIB	PII+III	No	No	7.5	4.6	6.9	No	Not applicable
13	MGMII	Chondrosarcoma	IIB	P II	Yes	Yes	5.5	6.0	5.8	No	Not applicable
14	MGMII	Renal clear cell carcinoma	/	PII+III	Yes	Yes	8.6	6.0	7.1	No	Not applicable
15	MGMII	hepatocellular carcinoma	/	PII+III	Yes	Yes	7.6	9.2	6.7	No	Not applicable
16	MGMII	Ewing sarcoma	IIB	PII+III	Yes	Yes	8.5	4.7	5.1	Two cycles	21
17	MGMII	Renal clear cell carcinoma	/	PII+III	Yes	Yes	4.5	3.5	7.3	No	Not applicable
18	MGMII	Ewing sarcoma	IIB	PII+III	Yes	Yes	11.8	7.0	8.5	Two cycles	29
19	MGMII	Giant cell tumor	/	PII+III	Yes	Yes	10.2	5.9	8.7	No	Not applicable
20	MGMII	Giant cell tumor	/	PII+III	Yes	Yes	7.5	3.0	5.0	No	Not applicable
21	MGMII	Chondrosarcoma	IIB	PII+III	Yes	Yes	6.2	4.7	5.6	No	Not applicable
22	MGMII	Chondrosarcoma	IIB	PII+III	Yes	Yes	8.2	6.3	9.8	No	Not applicable
23	MGMII	Osteosarcoma	IIB	PII+III	Yes	Yes	5.7	5.2	6.2	Two cycles	19
24	MGMII	Chondrosarcoma	IIB	PII+III	Yes	Yes	6.2	4.7	5.6	No	Not applicable
25	MGMII	Ewing sarcoma	IIB	PII+III	Yes	Yes	7.4	7.7	8.0	Two cycles	20

a According to Enneking and Dunham (15).

MGMII, modified Gibson and modified ilioinguinal.

### Surgical Approach Preference

The surgical approach was selected mainly based on the location and extent of the tumor. In our study, the main indication for use of MGMII approach during the period in question was that the tumor invaded both ischial body and ischiopubic ramus, or the ischial tuberosity was invaded. Since lower body mass index (BMI) was associated with lower soft tissue thickness, which favor surgical exposure, low BMI was a relative indication for use of MGMII. Meanwhile, the contraindications were pelvic tumors requiring type I resections and gluteus medius requiring resection due to tumor invasion. Moreover, if the osteotomy plane was higher than the greater sciatic notch level, the MGMII approach was not recommended due to potential impairment of hip abductors. Alternatively, the standard iliofemoral approach was used for the patients who required a type II resection of lateral acetabular tumors.

Based on this surgical approach preference, 25 patients received hemipelvic replacement surgery though an MGMII approach or an iliofemoral approach. All patients were divided into two groups according to different approaches, including 13 patients with an MGMII approach (MGMII group) and 12 patients with an iliofemoral approach (iliofemoral group). There was no significant difference in age (p = 0.423) and BMI (p = 0.472) between two groups. There was no significant difference in preoperative tumor size between the two groups [MGMII group vs. iliofemoral group, median of 6.1 (interquartile range, IQR, 5.7–7.7) vs. median of 6.6 (IQR, 5.9–7.9), p = 0.676]. However, the number of patients with tumor invasion of the posterior column of the acetabulum (p < 0.001) or ischial tuberosity (p < 0.001) was significantly more in the MGMII approach group than in the iliofemoral approach group (**Table 2**).

### Endoprosthesis Design and Fabrication

Preoperatively, apart from the routine plain radiography and single-photon emission CT or positron emission tomography/CT, 3D-CT and magnetic resonance imaging (MRI) were performed. We obtained the data and imported them into the Mimics V20.0 software (Materialise Corp., Leuven, Belgium) to enable image fusion and preoperative plan. According to the tumor-free margin, the surgical simulation and endoprosthesis design were undertaken. All prostheses were designed by our clinical team and fabricated by Beijing Chunlizhengda Medical Instruments Co., Ltd (Tongzhou, Beijing, China). Additional details related to the prosthesis design and application were illustrated in our previous study (31).

### Surgical Techniques

**Figure 1:**
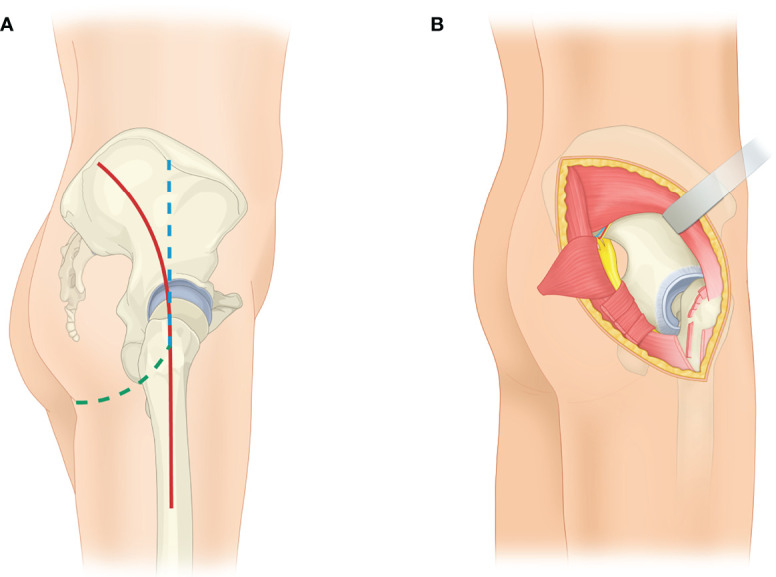
Schematic depiction of the modified Gibson approach. **(A)** The routine Gibson approach (red) is shown. Both proximal and distal part of the approach can be modified according to tumor involvement. If majority of the anteroposterior ilium is under exposure, the proximal part of the modified approach (blue) would be used. Meanwhile, if the whole ischial tubercle was involved, the distal part of the incision (green) would be modified to follow the gluteus fold. **(B)** The image shows the exposure to the outer surface of the innominate bone *via* a modified Gibson approach. The femoral neck osteotomy and femur upshifting have been proceeded to relax the muscles, vessels, and nerves. The origin of the gluteus minimus is released and retracted with preserved gluteus medius proximally. The insertion of the gluteus maximus is released, and the gluteus maximus is retracted posteriorly.

All operations were performed using a lateral floating position by the same senior surgeon (Chongqi Tu). Tranexamic acid was administered to reduce perioperative blood loss in both groups. All patients were given intravenous tranexamic acid based on their weight (15 mg/kg) to reduce perioperative blood loss. Specifically, a certain dose of tranexamic acid was administered before skin incision versus before wound closure. The modified Gibson approach (21) outlined the upper border of the muscle and arched slightly forward from a point 6 cm in front of the posterior superior spine to the greater trochanter and then turned posteriorly and followed the gluteus fold. In patients whom the anteroinferior ilium was involved, the incision superior to the greater trochanter would be similar to a straighter modified Gibson approach (**Figures 1A, B**) (32). After identifying the sciatic nerve and following it up to the greater sciatic notch, we exposed the joint capsule. The routine joint capsule incision, posterior hip dislocation, and femoral neck osteotomy were then performed to maneuver the limb location flexibly. The limb was upshifted and rotated externally to offer the space for elevating the gluteus minimus off the underlying bone from distal to proximal. The superior gluteal neurovascular bundle, exiting superior to the level of the sciatic nerve, was identified carefully with palpation of the superior gluteal artery. Sufficient bone surface to seat the patient-specific instruments should be ensured.

**Figure 2:**
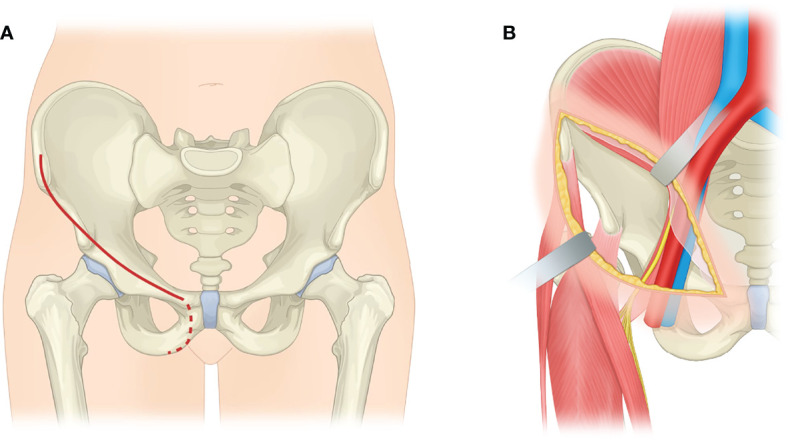
Schematic depiction of the modified ilioinguinal approach. **(A)** The ilioinguinal incision (red solid line) is shown. The incision can extend following the inferior pubic ramus (red dotted line) to exposed peri-obturator region, if needed. **(B)** The image shows the exposure to the inner surface of the innominate bone *via* a modified ilioinguinal approach. The iliopsoas is relaxed by upshifting the femur and is retracted to access the iliopectineal arch.

The modified ilioinguinal approach was utilized to expose the peri-obturator structures (**Figures 2A, B**). The incision was part of the ilioinguinal incision and usually begins at the point 2 cm posterior and 2 cm proximal to the anterior superior iliac spine, runs parallel and slightly above the inguinal ligament, and ends at the pubic tubercle. The incision can be extended proximally along the iliac crest and distally following the inferior pubic ramus according to the tumor-free margin (33). Identification of the femoral nerve, which is at the medial-deep aspect of the iliopsoas, is the key step.

Once osteotomy was undertaken, the sacrotuberous ligament, sacrospinous ligament, and other remaining soft tissues were dissected in order to facilitate the removal of the specimen. We consequently rotated the specimen internally and pulled it out to *via* the modified Gibson incision or the modified ilioinguinal approach.

The exposure process in patients who received the internal hemipelvectomy *via* routine iliofemoral approach with or without ilioinguinal approach and the implantation process of all patients have been illustrated in our previous study (31).

### Postoperative Management

Prophylactic intravenous (IV) antibiotics (1,500 mg IV cefazolin sodium) were given 30 min before catheterization. Prophylactic antibiotics were continued for 72 h postoperatively. Low molecular weight heparin (2,500 IU/day) was administered *via* subcutaneous injection postoperatively until the patient can move freely. Allogeneic transfusion was given postoperatively if Hb concentration fell below 7 g/dl, and no transfusion was given if Hb concentration >10 g/dl. For patients with Hb concentration of 7–10 g/dl, surgeons can decide whether transfusion is needed according to the presence or absence of symptoms of anemia (such as dyspnea or tachycardia) after volume replacement therapy. However, considering the huge trauma of surgery to patients and the effect of postoperative hemoglobin levels on incision healing, we prefer to transfuse this group of patients.

After surgery, the limb was immobilized in a neutral rotation, 15°–25° hip abduction, 15° hip flexion, and 15° knee flexion with arthrosis. Besides, all patients were encouraged to perform ankle pump exercises while they were administrated with patient-controlled analgesia (PCA). Within 3 days, overall hip muscle strength was evaluated with hip stability test and knee extension test to determine following personalized rehabilitation program (31).

In the MGMII group, the minor passive hip flexion and abduction training were administered 3 days postoperatively, and the gradual weight-bearing stance with walking aids was allowed 1 week postoperatively. The active hip flexion and abduction and single-leg stance started about 2 weeks postoperatively. Ambulation with walking aids was managed 3 weeks postoperatively. During the second month after the surgery, stance and walk with a cane was enabled in patients with satisfying performance in training. At the end of the second month, patients were encouraged to ambulate without canes and allowed to squat under supervision.

In the iliofemoral group, 1 week of in-bed training, 1 week of standing hip flexion with no weight bearing of the affected limb, 2 weeks of increasing weight-bearing on the affected limb, and 8 weeks of hip abduction, adduction, and extension training, and ambulation with walking aids were managed in the first 3 months. Thereafter, all patients were encouraged to walk without crutches and asked to start cross-leg and squat training, which lasted for 1 week (31).

Systematic clinical and radiological follow-up was conducted at 1, 2, and 3 months, then every 3 months for the first 2 years and six-monthly thereafter. All patients were followed for 2 years or longer; at a median follow-up interval of 41 months (IQR, 33.5–46 months), no patient was lost to follow-up.

### Primary, Secondary, and Third Endpoints

All main endpoints were independently assessed by a surgeon who was not involved in the patient’s care, thus eliminating the risk of assessor bias. Our primary endpoint of interest was whether the convenience and accuracy in terms of internal hemipelvectomy can be provided *via* an MGMII approach. The operation duration, blood loss, and packed red blood cells transfusion were assessed by chart review. The amount of bleeding was jointly calculated by the anesthesiologist and the surgical nurse and recorded in the medical record, and it was calculated as the amount of fluid in the reservoir tank of suction apparatus plus the amount of dressing bleed. The blood leakage volume of dressing, namely, blood soaking area of 10 × 10 cm, is 10 ml. In addition, the tumor size was represented by the tumor length plus width plus height divided by 3, which were measured by the last MRI before surgery. The specimens with tumor were sent to the laboratory for histopathological studies. Bone margins were assessed at the osteotomy, and soft tissue margins were assessed at the circumferential soft tissue. If the pathology report could not determine the surgical margins of bone or soft tissue or did not provide information about the minimal margins, the report and tissue sections were reviewed by a senior pathologist specializing in bone tumors. According to the R classification (34), residual tumors are referred to as R0, R1, or R2 [R0, no residual tumor, margin ≥ 1 mm; R1, no residual tumor (microscopic residual tumor), margin ≤ 1 mm; and R2, macroscopic residual tumor].

Our second endpoint was whether the MGMII approach benefited functional recovery. The hip-abductor muscle strength of both sides was measured in lateral decubitus position with a hand-held dynamometer (Model 01163; Lafayette Instrument, IN) at 2, 3, and 12 months postoperatively, and the abductor muscle strength ratio of the affected side to healthy side was recorded. The 1993 version of the Musculoskeletal Tumor Society (MSTS-93) and the Harris Hip scores (HHS) were evaluated (35). The limp score was recorded as 3 at non-limping, 2 with slight limping, and 1 with severe limping (**Table 3**).

**Table 3:** Comparison of intraoperative, endoprosthetic, and functional status following internal hemipelvectomy *via* two approaches.

	PISP group	GMII group	p-value
Operative duration (minutes)	Median 315 (IQR, 255–375)	Median 250 (IQR, 220–290)	0.036
Preoperative hemoglobin (g/L)	Median 128 (IQR, 121.75–138.25)	Median 118 (IQR, 114–135)	0.458
Blood loss (ml)	Median 2,400 (IQR, 1,950–3,000)	Median 1700 (IQR, 1350–2000)	0.012
PRBC transfusion (units)	Median 7.25 (IQR, 5.875–8.875)	Median 5.0 (IQR, 0.0–8.0)	0.137
Postoperative hemoglobin (g/L)	Median 100.5 (IQR, 97.25–102.75)	Median 108 (IQR, 102–113)	0.045
Tumor size (cm)	Median 6.6 (IQR, 5.9–7.9)	Median 6.1 (IQR, 5.7–7.7)	0.676
Hip abductor strength ratio 2 months postoperatively	Median 63.5% (IQR, 59.5%–68.5%)	Median 76% (IQR, 71%–78.5%)	0.003
Hip abductor strength ratio three months postoperatively	Median 77% (IQR, 71%–81.5%)	Median 92% (IQR, 89.5%–94%)	0.002
Hip abductor strength ratio 12 months postoperatively	Median 84% (IQR, 77.5%–87.5%)	Median 98% (IQR, 96.5%–98.5%)	0.002
MSTS-93	Median 24 (IQR, 22.5–26)	Median 29 (IQR, 27.5–29.5)	0.005
Harris hip score	Median 82 (IQR, 79.5–83.5)	Median 95 (IQR, 93–98)	0.002
Limp	Median 2 (IQR, 2–2)	Median 3 (IQR, 2–3)	0.008

PISP, posterior iliac and Smith–Peterson; GMII, Gibson and mini-ilioinguinal; PRBC, packed red blood cells; IQR, interquartile range.

Our third endpoint was short-term complications associated with the use of the two approaches. The assessor reviewed the patients’ record to assess the major complications, including delayed wound healing, deep infection, dislocation, and aseptic loosening.

### Statistical Analysis

The statistical analyses were performed using IBM SPSS Statistics software, version 25 (IBM SPSS, Armonk, NY, USA). The Wilcoxon signed-rank test was used to compare age, BMI, operation duration, blood loss, preoperative hemoglobin, postoperative blood transfusion, postoperative hemoglobin, hip abductor strength ratios, MSTS-93 score, HHS, and limp score of each group. The rates of posterior acetabular column involvement, ischial tuberosity bone destruction, and packed red blood cells transfusion were compared between the two groups using Fisher’s exact test. A p-value <0.05 was considered statistically significant.

## Results

### Surgery Convenience and Accuracy

R0 resection was achieved in all patients of both groups. However, the MGMII group was superior to the iliofemoral group in terms of surgery convenience and accuracy, which could be reflected in the operative duration and blood loss. The operative duration in the MGMII group, with a median value of 250 min (IQR, 220–290 min), was shorter than that in the iliofemoral group with a median value of 315 min (IQR, 255–375 min) (p = 0.036). Meanwhile, the intraoperative blood loss in the MGMII group, with a median value of 1,700 ml (IQR, 1,350–2,000 ml), is lower than that in the iliofemoral group with a median value of 2,400 ml (IQR, 1,950–3,000 ml) (p = 0.012). There was no significant difference between the two groups of patients in terms of preoperative hemoglobin (p = 0.458), postoperative packed red blood cells transfusion rate (p = 0.378), and postoperative blood transfusion (p = 0.137). However, patients in the MGMII group had slightly higher postoperative hemoglobin levels (median, 108; IQR, 102–113 g/L) than those in the iliofemoral group (median, 100.5; IQR, 97.25–102.75 g/L).

### Functional Recovery

Accelerated and superior functional recovery was obtained *via* the MGMII approach. The hip abductors are stronger in the MGMII group at all three time points after surgery (MGMII group vs. iliofemoral group):

- Two months: median of 76% (IQR, 71%–78.5%) vs. median of 63.5% (IQR, 59.5%–68.5%), p = 0.003
- Three months: median of 92% (IQR, 89.5%–94%) vs. median of 77% (IQR, 71%–81.5%), p = 0.002
- Twelve months: median of 98% (IQR, 96.5%–98.5%) vs. median of 84% (IQR, 77.5%–87.5%), p = 0.002

At the most recent follow-up examination, patients in the MGMII group received better functional restoration with a median MSTS-93 score of 29 (IQR, 27.5–29.5) and median HHS of 95 (IQR, 93–98), in comparison to patients in the iliofemoral group, with a median MSTS-93 score of 24 (IQR, 22.5–26) (p = 0.005) and a median HHS of 82 (IQR, 79.5–83.5) (p = 0.002). Less patients encountered limp in the MGMII group with a higher limp score [MGMII group vs. iliofemoral group, median of 3 (IQR, 2–3) vs. median of 2 (IQR, 2–2); p = 0.008].

### Complications

No complications were observed in the MGMII group. Three patients in the iliofemoral group encountered delayed wound healing, and all these patients underwent debridement and closure procedures; after 1 month, their wounds healed. One patient in the iliofemoral group developed deep infection 1 week after the surgery, which was successfully treated by staged debridement and intravenous antibiotics. No further amputation or revision surgery was performed.

## Discussion

**Figure 3:**
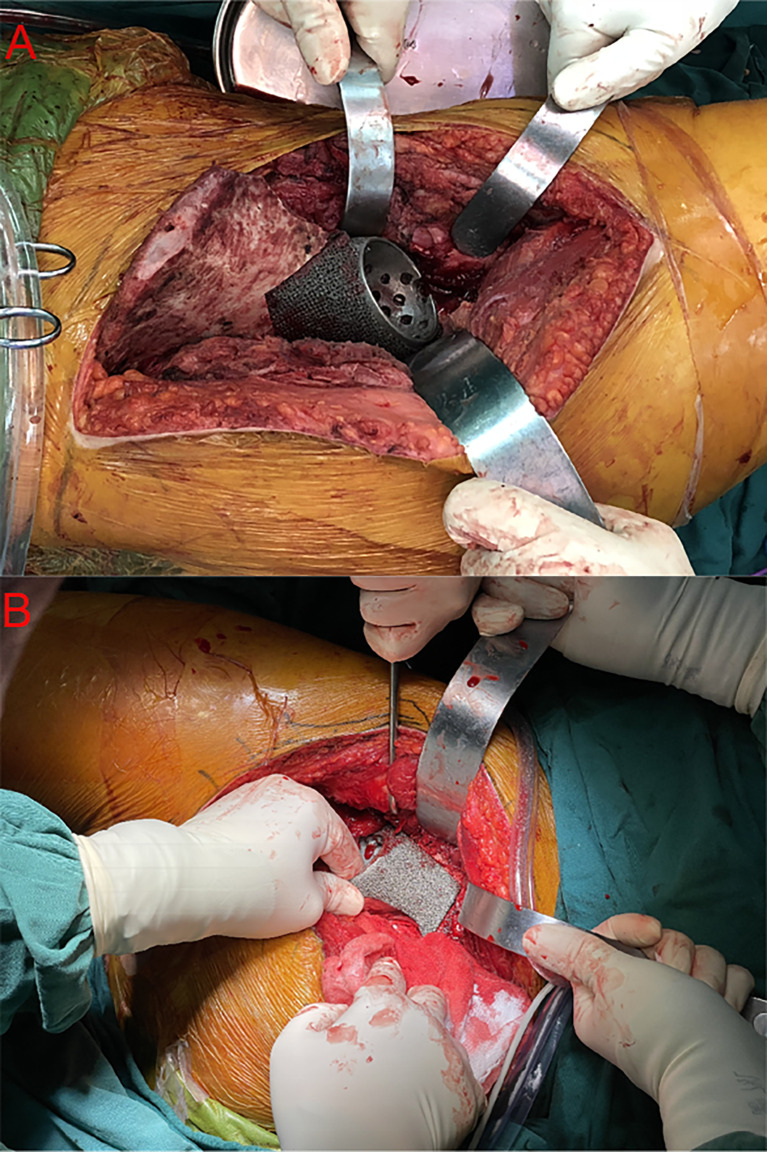
Intraoperative photograph of the approaches: The intraoperative images show intimate contact between the host bone and 3D-printed custom-made endoprosthesis *via* the iliofemoral approach in patient 6 **(A)** and the modified Gibson approach in patient 17 **(B)**.

**Figure 4:**
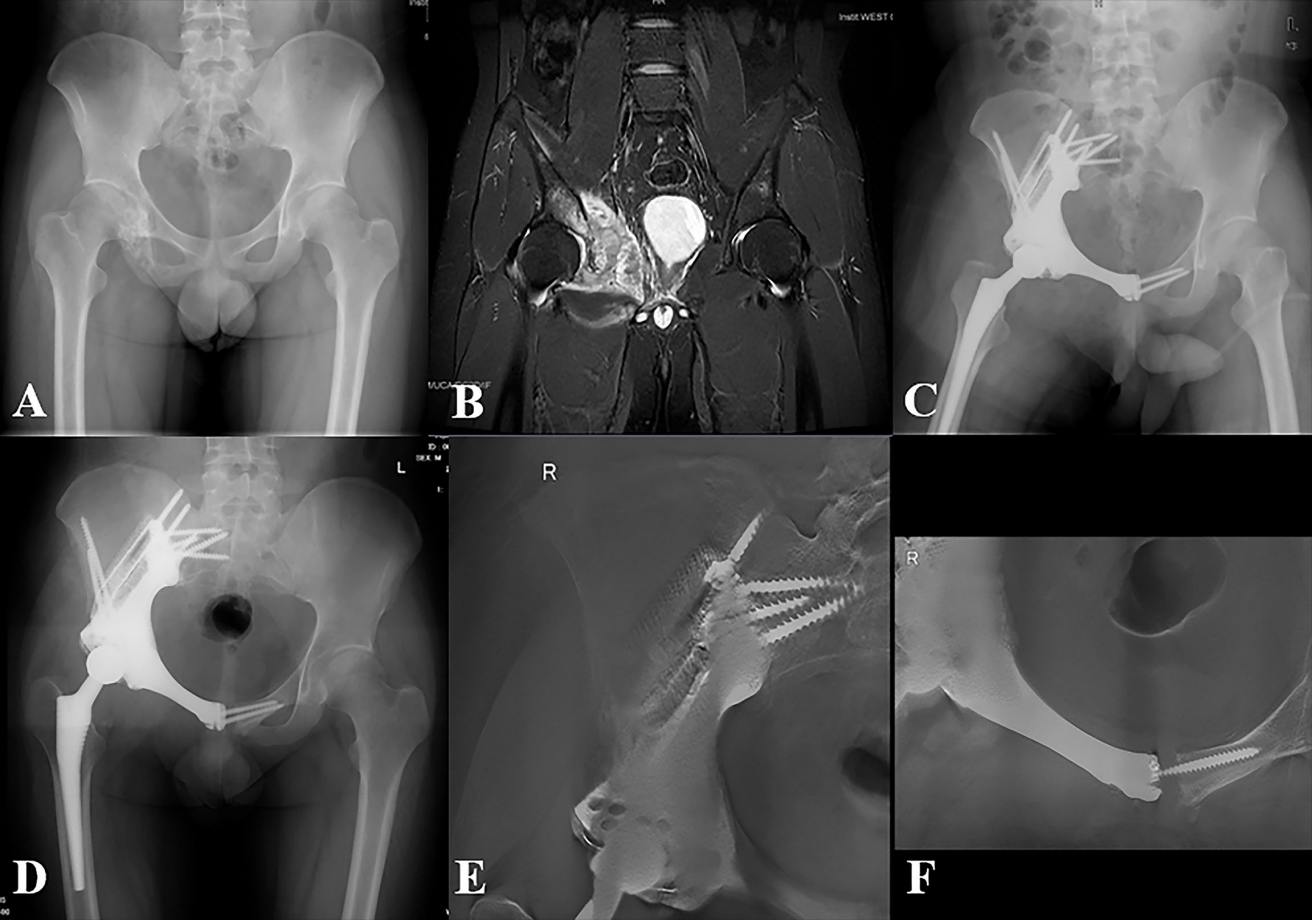
Representative case of the MGMII group. **(A)** Preoperative X-ray of the pelvis of patient 16. **(B)** Preoperative MRI of the pelvis. **(C)** X-ray of the pelvis taken at 3 days after surgery. **(D)** X-ray of the pelvis taken at 10 months after surgery. **(E, F)** T-SMART taken at 10 months after surgery showed excellent osseointegration.

The iliofemoral approach and its modifications have been widely accepted in type II+III resection to provide wide and direct visualization to majority of innominate bone (10, 11, 16, 36). However, this massive approach detaches the normal gluteus and iliacus from the iliac bone excessively, leading to prolonged rehabilitation duration and common limping gait (10, 11, 13). Comparably, the MGMII approach offers sufficient exposure for type II+III resection with minimized disruption to periacetabular muscles to enable early rehabilitation (**Figures 3A, B**). In addition, with a series of 3D-printed custom-made patient-specific instruments and endoprostheses, precise resection and reconstruction are accessible (**Figures 4A, B**). For patients with type II + III tumors involving the posterior column of the acetabulum, especially the ischial tuberosity, the advantages of the MGMII approach used in hemipelvic replacement with 3D-printed custom-made endoprosthesis were mainly reflected in the following four aspects.

**Figure 5:**
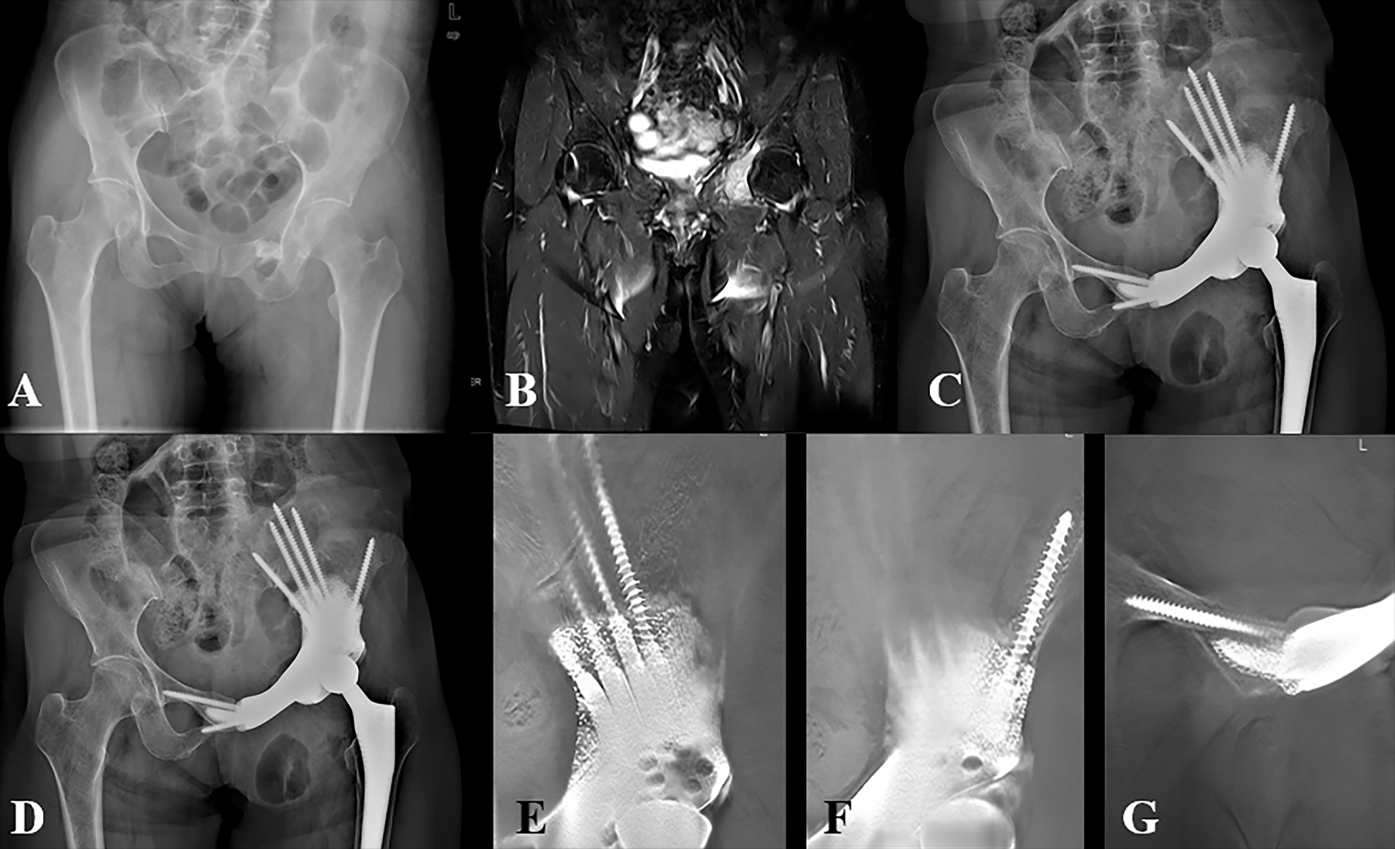
Representative case of the iliofemoral group. **(A)** Preoperative X-ray of the pelvis of patient 11. **(B)** Preoperative MRI of the pelvis. **(C)** X-ray of the pelvis taken at 3 days after surgery. **(D)** X-ray of the pelvis taken at 30 months after surgery. **(E–G)** T-SMART taken at 6 months after surgery showed excellent osseointegration.

First, the operation is facilitated with accuracy *via* the MGMII approach. Since en bloc resection and strictly adhering to the tumor-free principle are the foremost issues in bone oncology, full exposure of the tumor site is of great importance. However, for some type II+III patients, due to the occlusion of the tumor, the iliofemoral approach may not be able to effectively expose the posterior column of the acetabulum, especially the ischial tuberosity (**Figures 5A, B**). During the operation, the forward force applied to the tumor in order to completely remove the tumor acts with the tension of the ligaments and attached muscles at the ischial tuberosity, which easily leads to the rupture of the tumor at the ischial tuberosity. In addition, patients with pathological fracture of the ischial tuberosity would further add to the difficulty of resecting tumor *via* a sole-incision approach. By contrast, the MGMII approach offers considerable local exposure *via* two relatively shallow incisions: the modified ilioinguinal approach is beneficial to the exposure of the anterior acetabular, obturator foramen, and pubic symphysis. Meanwhile, the modified Gibson approach provided an excellent exposure in terms of the entire acetabular, body of ischium, ischial tuberosity, and a part of ramus of ischium. By the wide exposure of these complicated structures, operation can be facilitated with accurate ablation under direct vision. Theoretically, a two-incision approach is expected to be more time consuming to accomplish en bloc type II+III resection, whereas it saves time by providing direct access to surgical field and avoiding extensive neurovascular structure separation. The reduced operation duration can lessen intraoperative blood loss, which is majorly caused by the continuous diffuse blood oozing from the massive wound. In our study, the MGMII approach exhibited its advantages in bleeding and trauma control for specific patients, in line with the ERAS protocols.

Second, accelerated and superior functional recovery is obtained *via* the MGMII approach. The ERAS protocols emphasize reducing surgical trauma to benefit early mobilization and functional recovery (37, 38). Comparing to the iliofemoral group, the absence of detachment and reattachment of hip abductor mechanism in the MGMII group leads to less extensive muscle swelling and therefore facilitate regaining the ability to stabilize hip joint and accelerate the rehabilitation program. Consequently, the abductor strength grows faster to the close normal level in the MGMII group during the first 2 months, while the abductor strength in the iliofemoral group requires 3 months or more to achieve similar level, or, worse, some patients’ abductor strength get impaired during whole follow-up duration. The extensive scar tissue around the periacetabular muscles might contribute to such strength impairment. Apart from rehabilitation factor, the surgical technique can also impact functional recovery. While reestablishing periacetabular muscles in the iliofemoral group, only superficial muscle fibers are reattached to the iliac crest. A majority of the deep muscle fibers are in a state of soft tissue failure due to the linear muscle reconstruction (39). Then, their further disuse-atrophy and fatty infiltration might cause cosmetic problem, hip pain, and function decline. As a result, patients in the MGMII group regain superior lower limb function and better gait performance, comparing to the iliofemoral group and most of previous studies (6, 14, 40).

Third, the MGMII group achieved a reduced risk of postoperative complications. No complication was observed in the MGMII group, while several complications occurred in the iliofemoral group. All complications can postpone rehabilitation program and therefore negatively impact implementation and validity of ERAS strategies. Previously, wound complication has been reported with an incidence range 10%–40% following internal hemipelvectomy *via* iliofemoral approach (4, 8, 10). The reasons are considered to be the intraoperative skin ischemia caused by constant retraction and impaired blood supply resulting from longitudinal incision through the groin area (41). Besides, given an extralong incision in the iliofemoral approach, early excessive rehabilitation can also result in wound dehiscence. However, the MGMII approach, avoiding entering the core zone of groin longitudinally, is believed to mitigate anterior vascular lesions (42). In addition, infection is common *via* the iliofemoral approach previously (10). The surgical-induced intervals are prone to emerge dead space and further infection. Compared with the MGMII approach, the iliofemoral approach requires exposure of both surfaces of the ilium and therefore leads to a larger dead space to imperil infection-free survival (5). Moreover, no dislocation was encountered in both groups even though we adopted early mobilization strategy. Apart from a carefully designed acetabular orientation, precise resection and reconstruction procedures, and a constrained acetabular liner with increased anteversion during implantation (31), the good reactivation of the gluteus and restoration of the articular capsule and a step-by-step rehabilitation program are also considered advantaging dislocation prevention.

Fourth, the MGMII approach has a high degree of application flexibility, which enables the surgeon to adapt to particular circumstances. For example, if the tumor was mainly located deep in the pelvic space, the modified ilioinguinal incision could be appropriately extended. Conversely, the modified Gibson approach could be adjusted by properly tuning the direction and length of the incision, when the tumor mainly invaded lateral margins of the acetabulum. Additionally, after resecting the tumor, the specimens can be pulled out though different incisions, which also provided good selectivity for the operation.

This study has certain limitations. First, even though strict inclusion and exclusion criteria were formulated, there was still some heterogeneity of the included patients like the different tumor types, tumor metastases, and the use of chemotherapy. Second, although whether the gluteus medius was detached or not was the main variable, the resection level and soft tissue excision varied and were dependent on the individual tumor, and as such, it was difficult to account for individual variables of the unique resections. Finally, the retrospective study, non-randomized and apparently not blinded, is associated with the selection bias; hence, prospective randomized studies are still needed to verify the roles of the MGMII approach in hemipelvic replacement.

## Conclusions

Our study showed that the iliofemoral and MGMII approaches are both reliable for patients with type II + III tumors. However, the MGMII approach can better expose the posterior column of the acetabulum, especially the ischial tuberosity, which is beneficial for avoiding tumor rupture during resection. The MGMII approach also helps to preserve residual muscle function, such as the origin of the gluteus medius while ensuring the extent of resection.

## Data Availability Statement

The original contributions presented in the study are included in the article/supplementary material. Further inquiries can be directed to the corresponding authors.

## Ethics Statement

The studies involving human participants were reviewed and approved by the Ethics Committee of West China Hospital. Written informed consent to participate in this study was provided by the participants’ legal guardian/next of kin.

## Author Contributions

LM and CT designed the experiments; XH and ML prepared the manuscript; XH, JW, and LL performed the experiments; LL analyzed the data; CT revised the manuscript; and all authors read the manuscript and approved the submission.

## Funding

The institution of one or more of the authors has received, during the study period, funding from Chengdu science and technology project (2017-CY02- 00032-GX), 8122 Project, Qingdao Research Institute of Sichuan University (20GZ30301), and the Fundamental Research Funds for the Central Universities (2021SCU12010).

## Conflict of Interest

The authors declare that the research was conducted in the absence of any commercial or financial relationships that could be construed as a potential conflict of interest.

## Publisher’s Note

All claims expressed in this article are solely those of the authors and do not necessarily represent those of their affiliated organizations, or those of the publisher, the editors and the reviewers. Any product that may be evaluated in this article, or claim that may be made by its manufacturer, is not guaranteed or endorsed by the publisher.
